# Adolescent affective symptoms and mortality

**DOI:** 10.1192/bjp.2018.90

**Published:** 2018-07

**Authors:** Gemma Archer, Diana Kuh, Matthew Hotopf, Mai Stafford, Marcus Richards

**Affiliations:** 1 Medical Research Council Unit for Lifelong Health and Ageing, University College London, UK; 2 Institute of Psychiatry, Psychology & Neuroscience, King's College London, UK, and South London and Maudsley NHS Foundation Trust, UK; 3 Medical Research Council Unit for Lifelong Health and Ageing, University College London, UK

**Keywords:** Mortality, depression, adolescent, cohort studies, affective disorders

## Abstract

**Background:**

Little is known about the relationship between adolescent affective problems (anxiety and depression) and mortality.

**Aims:**

To examine whether adolescent affective symptoms are associated with premature mortality, and to assess whether this relationship is independent of other developmental factors.

**Method:**

Data (*n* = 3884) was from Britain's oldest birth cohort study – the National Survey of Health and Development. Adolescent affective symptoms were rated by teachers at ages 13 and 15 years: scores were summed and classified into three categories: mild or no, moderate and severe symptoms (1st–50th, 51st–90th and 91st–100th percentiles, respectively). Mortality data were obtained from national registry data up to age 68 years. Potential confounders were parental social class, childhood cognition and illness, and adolescent externalising behaviour.

**Results:**

Over the 53-year follow-up period, 12.2% (*n* = 472) of study members died. Severe adolescent affective symptoms were associated with an increased rate of mortality compared with those with mild or no symptoms (gender adjusted hazard ratio 1.76, 95% CI 1.33–2.33). This association was only partially attenuated after adjustment for potential confounders (fully adjusted hazard ratio 1.61, 95% CI 1.20–2.15). There was suggestive evidence of an association across multiple causes of death. Moderate symptoms were not associated with mortality.

**Conclusions:**

Severe adolescent affective symptoms are associated with an increased rate of premature mortality over a 53-year follow-up period, independent of potential confounders. These findings underscore the importance of early mental health interventions.

**Declaration of interest:**

None.

Adolescent affective symptoms are associated with a range of negative adult outcomes, including low levels of educational attainment,^1^^,^^2^ negative health behaviours^3^^–^^5^ and adult mental health problems,^6^ which in turn are associated with excess mortality across multiple causes of death.^7^^–^^10^ It is therefore plausible that adolescent affective symptoms are themselves an important risk factor for premature mortality; however, to our knowledge, only two studies have examined this. Lee *et al.*,^11^ demonstrated an association between high adolescent trait anxiety and non-accidental mortality up to age 53 years in the Medical Research Council's National Survey of Health and Development (NSHD; also known as the 1946 British birth cohort study); however, analyses were adjusted for gender only and anxiety was assessed by a single-item measure that classified over 45% of the sample as a ‘case’. Likewise, Jokela *et al.*^12^ used a relatively weak exposure measure, capturing symptoms that were not a direct measure of internalising disorder. Nevertheless, they found an association between this measure and premature mortality up to age 46 years in the 1958 British birth cohort, although the association was not robust to adjustment for confounders, including childhood social class, cognition and externalising behaviours. We therefore used data from the NSHD when followed to age 68 years, to i. examine whether adolescent affective symptoms (rated in detail by teachers) were associated with all-cause mortality; ii. to assess whether this association was independent of numerous potential confounders, including parental social class, childhood health and cognition, and concurrent mental health problems; and iii. to examine whether associations were observed across multiple causes of death.

## Method

### Data

The NSHD is Britain's oldest birth cohort study. A socially stratified sample of 5362 (2547 female, 2815 male) singleton births were selected for follow-up from all births occurring during a single week in March 1946. The cohort has been followed up 24 times across the life course, most recently at ages 68–69 years.^13^ Participation rates have been high throughout the study, and comparisons between the NSHD and census data have shown that the remaining cohort sample is broadly representative of all native-born adults in the general population.^14^^,^^15^

Ethical approval was granted by the National Research Ethics Service Committee London Queen Square (14/LO/1073) and by the Scotland A Research Ethics Committee (14/SS/1009). All study members gave signed informed consent.

### Mortality

Mortality data were obtained from study records and linked with National Health Service Central Registry data. Cause of death was coded according to the ICD-9 and ICD-10.^16^^,^^17^ We focused on main causes of death: cardiovascular disease (ICD-9 codes 401–454 and ICD-10 codes 110–189), cancers (ICD-9 codes 140–239 and ICD-10 codes C00–C97) and externalising causes (violent, accidental and suicidal deaths; ICD-9 codes 800–994, 1800–1869 and 1880–1999 and ICD-10 codes S00–X99); however, all other causes were identified. Follow-up time was from age 15 years to mortality, until censored owing to emigration (*n* = 284) or to the end of October 2014.

### Adolescent affective symptoms

Affective symptoms were assessed by teachers, using a forerunner of the Rutter B questionnaire,^18^ which predates the introduction of diagnostic criteria. At ages 13 and 15 years, teachers were asked to rate the study member's behaviour compared with other children in the class, using a three-category response scale: more than, the same or less than other children. The questionnaires were subjected to exploratory factor analysis, which identified three distinct factors relating to emotional problems, externalising behaviour (conduct problems) and self-organisation.^19^^,^^20^ Ten items were indicators of emotional problems: anxious, always tired and washed out, frightened of rough games, extremely fearful, avoids attention, usually gloomy and sad, timid child, unable to make friends, diffident about competing and unduly miserable or worried about criticism; model fit indices of measurement invariance indicated excellent fit, suggesting adequate reliability.^20^ The factor scores of Xu *et al.*^20^ for emotional problems were standardised with *z*-scores at ages 13 and 15 years, and then summed to create a single measure of adolescent affective symptoms.

We generated a three-category ordinal variable based on previously used cut-off points:^2^^,^^19^ study members between the 1st and 50th percentiles were classified as having mild or no symptoms, those between the 50th and 90th percentiles were classified as having moderate symptoms and those between the 91st and 100th percentiles were classified as having severe symptoms. The cut-off point for severe symptoms is in keeping with 12-month prevalence rates for adolescent anxiety and depressive disorders reported in samples in Europe and the USA^21^ (6.9–9.5% for anxiety and 2.1–3.4% for depressive disorders).

### Confounding factors

Potential confounders were identified *a priori* as childhood social, psychological and physical health factors associated with lifetime mental health and mortality in other studies, including the NSHD.^22^^–^^24^ These were prospectively measured childhood social class (based on the occupation of the study member's father at age 11 years, or if this was unknown, at age 4 or 15 years, and coded into six groups according to the Registrar-General's classification), childhood cognition at age 8 years (derived from tests designed by the National Foundation for Education Research and described in detail elsewhere^25^), teacher-rated adolescent externalising behaviour at ages 13–15 years (derived from factor analysis by Xu *et al.*^20^), birth weight (in kilograms, obtained from birth records), childhood sickness absence (0–4 weeks, 4–10 weeks and 10+ weeks, obtained from school records spanning ages 6–12 years) and childhood hospital admission at ages 0–5 years, 6–10 years and 11–15 years (obtained from parental interviews, school attendance records, medical examinations and hospital in-patient records). Schizophrenia was ascertained by questionnaire, interview, and hospital and general practitioner contact data up to age 43 years.^26^ Health behaviours and other adult psychiatric or psychological problems^27^ were not included as potential confounders because we considered these variables to be potential mediators on the causal pathway.

### Analyses

We used Kaplan–Meier graphs to compare the survival probability of those with mild or no, moderate and severe affective symptoms over the follow-up period, and tested the equality of survival curves with a log-rank test. Cox proportional hazards models were used to investigate the relationship between adolescent affective symptoms and all-cause mortality rates. Models were first adjusted for gender. The gender-adjusted hazard ratios for affective symptoms were then adjusted for each potential confounder in turn, with childhood sickness absence and hospital admissions grouped to represent physical health. A further model included all variables. The analyses were repeated using competing-risks analyses, to examine whether associations between affective symptoms and mortality were observed across different causes of death (cardiovascular, cancer, externalising and all other causes).

Gender interactions were tested using joint Wald tests; however, there was no evidence that associations differed in males and females (*P* = 0.86 for all-cause mortality and *P* = 0.19–0.54 for cause-specific mortality).

The proportional hazards assumption was checked by including an interaction term between log-time and affective symptoms. Associations were robust to sensitivity analyses, which included the use of a less stringent cut-off point to classify severe symptoms (84th–100th percentile) (see supplementary Table 1, available at https://doi.org/10.1192/bjp.2018.90), and the exclusion of people with schizophrenia (*n* = 24) to examine whether associations were driven by concurrent mental disorder.

### Sample

Eligible participants included all those who had complete data on affective symptoms at ages 13 and 15 years and who were linked with the National Health Service Central Register. Of the original birth cohort (*n* = 5362), 250 died and 838 refused participation in the study, emigrated or were unable to be traced before age 15 years. A further 342 had missing data on affective symptoms at ages 13–15 years, and 48 had non-linked mortality data, leaving 3884 study members in the analytical sample. To minimise data loss, multiple imputation with chained equations was used to impute missing data on the following covariates: birth weight (*n* = 17, 0.4%), childhood social class (*n* = 55, 1.4%), childhood cognition (*n* = 165, 4.2%) and childhood sickness absence (*n* = 647, 16.7%).^28^^,^^29^ The imputation analyses contained all study variables, including factors that have been shown to predict non-response in the NSHD, such as manual social class and poor childhood cognition.^14^ Analyses were run across 20 imputed data-sets. On visual inspection, the imputed results were very similar to those using observed values (see supplementary Table 2 for non-imputed results, and supplementary Fig. 1 for fully adjusted survival curves based on non-imputed data; the Kaplan–Meier function is not currently supported for imputed data). All analyses were carried out in STATA version 13.1.

## Results

Table 1 shows the characteristics of the original (non-imputed) data and imputed study sample were similar. Mean follow-up for mortality after age 15 years was 48.8 years (range 3.4–53.0), with a total of 189 609 person-years and 472 deaths. Males had a slightly higher mortality rate than females (2.75 compared with 2.21 per 1000 person-years, respectively). For both genders, the most common cause of death was cancer, followed by cardiovascular disease and externalising causes (violent, accidental or suicidal deaths). All other causes of death comprised diseases of the respiratory system (33.0%), digestive system (23.0%), nervous system (16.5%) and all other miscellaneous causes (27.5%). Affective symptoms were more commonly reported in females than males, with 10.5% of females and 7.6% of males rated as having severe symptoms.

**Table 1:** Descriptive characteristics of the original (non-imputed) data and imputed study sample; *n* = 3884 (2016 males and 1868 females).

	Non-imputed		Imputed		
	*N*	All, %	All, %	Males, %	Females, %
Mortality					
All-cause	472	12.2	12.2	13.3	10.9
Cause-specific mortality					
Cancer	206	43.6	43.6	41.0	47.1
Cardiovascular disease	117	24.8	24.8	26.5	22.6
Externalising causes	58	12.3	12.3	14.2	9.8
Other^a^	91	19.3	19.3	18.3	20.6
Affective symptoms					
None/mild	1942	50.0	50.0	53.9	45.8
Moderate	1592	41.0	41.0	38.4	43.7
Severe	350	9.0	9.0	7.6	10.5
Childhood social class					
Professional	220	5.8	5.7	5.7	5.7
Intermediate	703	18.4	18.3	19.2	17.4
Skilled, non-manual	572	14.9	14.9	14.6	15.2
Skilled, manual	1314	34.3	34.3	34.0	34.7
Partly skilled	760	19.9	19.9	19.4	20.4
Unskilled	260	6.8	6.8	7.1	6.5
Birth weight, kg					
Mean (s.d.)	3867	3.40 (0.51)	3.40 (0.51)	3.48 (0.52)	3.32 (0.50)
Childhood sickness absence, weeks					
0–4	1680	51.9	52.7	53.5	51.8
4–10	1183	36.6	36.4	36.3	36.5
10+	374	11.6	10.9	10.2	11.7
Childhood hospital admissions, years					
0–5	255	6.6	6.6	7.1	5.9
6–10	519	13.4	13.4	14.3	12.3
11–15	210	5.4	5.4	6.1	4.7

Total *N* varies for childhood social class, birth weight and childhood sickness absence because of missing data.

a. Diseases of the respiratory system (33%), digestive system (23%), nervous system (16.5%) and all other causes (27.5%).

**Fig. 1:**
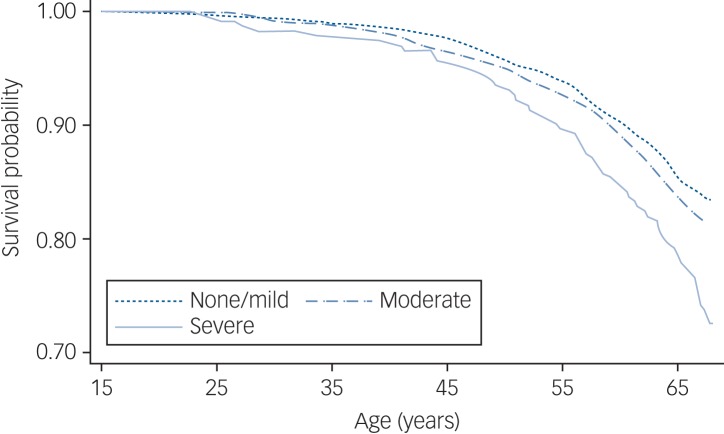
Gender-adjusted Kaplan–Meier survival curves for all-cause mortality by adolescent affective symptoms, based on 472 deaths (*n* = 3884).

Fig. 1 shows that study members rated with severe adolescent affective symptoms had the lowest survival probability throughout the follow-up period, followed by those with moderate and those with mild or no symptoms (the log-rank test showed that there was a difference in survival curves: χ^2^(2) = 14.1, *P* < 0.001). Markedly, the survival curve for severe symptoms continued to diverge for the duration of the follow-up period; the proportional hazards assumption was not violated (*P* = 0.58). By age 68 years, 18.6% of the severe group had died compared with 10.8% in the mild or no problem group.

Table 2 shows the association between adolescent affective symptoms and all-cause mortality, adjusted for potential confounders. After adjustment for gender, severe affective symptoms were associated with a higher rate of mortality compared with those with mild or no symptoms (hazard ratio 1.76, 95% CI 1.33–2.33). Childhood social class, cognition, birth weight, sickness absence and hospital admissions all slightly attenuated the association. Adolescent externalising behaviour was a weak negative confounder, whereas birth weight had no explanatory role. After adjustment for all potential confounders, the association remained but was partially attenuated (hazard ratio 1.61, 95% CI 1.20–2.15). The exclusion of people with schizophrenia did not alter the results (hazard ratio 1.58, 95% CI 1.18–2.13). The hazard ratios for moderate compared with mild or no symptoms were only slightly raised across all analyses and these associations were not statistically significant.

**Table 2:** Hazard ratios for the association between affective symptoms at age 13–15 years and all-cause mortality (15–67 years), based on 472 deaths and 20 imputations (*n* = 3884).

Model	Affective symptoms	Hazard ratio (95% CI)	Test for linear trend (*P* value)
Adjusted for gender	None/mild	1.0	<0.001
	Moderate	1.15 (0.95–1.40)	
	Severe	1.76 (1.33–2.33)**	
Adjusted for gender and childhood social class	None/mild	1.0	<0.01
	Moderate	1.12 (0.92–1.37)	
	Severe	1.67 (1.26–2.21)**	
Adjusted for gender and cognition at age 8 years	None/mild	1.0	<0.01
	Moderate	1.12 (0.92–1.37)	
	Severe	1.63 (1.23–2.17)*	
Adjusted for gender and externalising behaviour at age 13–15 years	None/mild	1.0	<0.001
	Moderate	1.18 (0.97–1.43)	
	Severe	1.81 (1.37–2.40)**	
Adjusted for gender and birth weight	None/mild	1.0	<0.001
	Moderate	1.15 (0.95–1.40)	
	Severe	1.76 (1.33–2.33)**	
Adjusted for gender, sickness absence at age 6–10 years and hospital admissions at ages 0–6, 6–10 and 11–15 years	None/mild	1.0	<0.01
	Moderate	1.14 (0.94–1.39)	
	Severe	1.64 (1.23–2.18)*	
Adjusted for all	None/mild	1.0	<0.01
	Moderate	1.13 (0.93–1.37)	
	Severe	1.61 (1.20–2.15)*	

* *P* < 0.05, **P < 0.001.

Table 3 shows associations between affective symptoms and mortality, by cause of death. After adjustment for gender, severe affective symptoms were associated with an increased rate of cardiovascular mortality (subdistribution hazard ratio 1.92, 95% CI 1.11–3.32) and other causes of death (subdistribution hazard ratio 2.65, 95% CI 1.47–4.80), and showed a borderline association with cancer mortality (subdistribution hazard ratio 1.52, 95% CI 1.00–2.31), compared with those with mild or no symptoms. There was no evidence of an association between severe affective symptoms and externalising causes of death; however, reliable estimates could not be generated in this subgroup as there were only four from externalising causes deaths (one of which was suicide) among those with severe symptoms.

**Table 3:** Subdistribution hazard ratios for the association between affective symptoms at age 13–15 years and cause-specific mortality (15–67 years), based on 20 imputations (*n* = 3884).

		Subdistribution hazard ratio (95% CI)	
Cause of death	Affective symptoms	Gender adjusted	Fully adjusted^a^
Cancer (206 deaths)	None/mild	1.0	1.0
	Moderate	0.94 (0.70–1.26)	0.94 (0.69–1.26)
	Severe	1.52 (1.00–2.31)	1.49 (0.97–2.28)
Cardiovascular disease (117 deaths)	None/mild	1.0	1.0
	Moderate	1.24 (0.83–1.84)	1.16 (0.79–1.72)
	Severe	1.92 (1.11–3.32)*	1.65 (0.93–2.91)
Externalising causes (58 deaths)	None/mild	1.0	1.0
	Moderate	1.25 (0.73–2.14)	1.28 (0.74–2.23)
	Severe	0.85 (0.29–2.47)	0.89 (0.31–2.56)
Other^b^ (91 deaths)	None/mild	1.0	1.0
	Moderate	1.55 (0.99–2.44)	1.44 (0.91–2.27)
	Severe	2.65 (1.47–4.80)*	2.03 (1.07–3.85)*

a. Adjusted for gender, childhood social class, cognition at age 8 years, externalising behaviour at age 13–15 years, birth weight, sickness absence at age 6–10 years and hospital admissions at ages 0–6, 6–10 and 11–15 years.

b. Diseases of the respiratory system (33%), digestive system (23%), nervous system (16.5%) and all other causes (27.5%).

* *P* < 0.05.

After adjustment for all potential confounders, an association remained between severe affective symptoms and other causes of death (subdistribution hazard ratio 2.03, 95% CI 1.07–3.85), but all other associations were attenuated to non-significance. The gender-adjusted subdistribution hazard ratios for cardiovascular mortality were attenuated by childhood social class (subdistribution hazard ratio 1.76, 95% CI 1.01–3.06), cognition (subdistribution hazard ratio 1.61, 95% CI 0.92–2.82), and childhood sickness absence and hospital admissions (subdistribution hazard ratio 1.82, 95% CI 1.06–3.16), whereas the gender-adjusted subdistribution hazard ratios for other causes were attenuated only by cognition (subdistribution hazard ratio 2.31, 95% CI 1.26–4.24) and childhood sickness absence and hospital admissions (subdistribution hazard ratio 2.21, 95% CI 1.20–4.05) (see supplementary Table 3). Estimates for cancer and externalising causes did not change with these adjustments (not shown).

## Discussion

In this large, UK population-based cohort, we found that severe adolescent affective symptoms were associated with a 61% increase in premature mortality over a 53-year follow-up period compared with those who had mild or no symptoms (hazard ratio 1.61, 95% CI 1.20–2.15, *P* < 0.05; equivalent to an additional 66 deaths per 1000 people). Notably, this relationship persisted for the duration of the follow-up period and was independent of a wide range of potential confounders, including parental social class, childhood hospital admissions, sickness absence and cognition, and adolescent externalising behaviour. There was also evidence to suggest that associations were observed across multiple causes of death.

### Comparison with other studies

Our study is the most comprehensive examination of the relationship between adolescent affective symptoms and premature mortality to date; we build on existing studies by extending mortality follow-up by at least 15 years, providing more rigorous control for potential confounding factors and using a stronger measure of affective problems.^11^^,^^12^ The findings are largely consistent with two previous studies examining the association between adolescent affective symptoms and mortality. Lee *et al.*^11^ and Jokela *et al.*^12^ both demonstrated gender-adjusted associations between adolescent affective symptoms and greater risk of premature mortality in population-based samples over a long period of follow-up. Jokela *et al.*^12^ found that associations were attenuated to non-significance after adjustment for adolescent externalising behaviour and other covariates such as cognitive ability and father's social class. This could be partly attributed to the study's measure of exposure (the ‘under-reaction’ dimension of the Bristol Social-Adjustment Guide^30^), which the author's note, could be considered related to, but not a direct measure of internalising. Furthermore, our study extends the follow-up period of Jokela *et al.*^12^ by almost 20 years, which could be important because causes of death vary by age; for instance, deaths from cancer and cardiovascular disease are more common at older ages compared with deaths from externalising causes.^31^ In the NSHD, Lee *et al.*^11^ found that high adolescent anxiety was associated with higher rate of non-accidental mortality after age 25 years, but a lower rate of accidental mortality before age 25 years. In keeping with Jokela *et al.*,^12^ we found no evidence of a protective effect of affective symptoms in our analyses. Lee *et al.*^11^ used a single-item measure of anxiety that classified over 50% of study members as a ‘case’, which may help to explain this discrepancy.

There is substantial continuity between depression experienced in adolescence and depression in adulthood;^6^^,^^32^ therefore, it is likely that the observed associations are, at least in part, a result of an accumulation effect. Equally, however, affective symptoms in adolescence may be especially detrimental because of their effect on educational attainment^1^^,^^2^ and subsequent negative social, psychological and behavioural factors.^1^^–^^3^^,^^6^ Because these factors are wide-ranging, it is understandable that we observed associations with mortality across several different causes of death. In particular, we found associations with deaths from cancers and cardiovascular disease after adjustment for gender; although the strongest associations were with deaths from other causes (predominantly diseases of the respiratory, digestive and nervous systems), which held after full adjustment for confounders. We did not have adequate power to examine the association between affective symptoms and deaths from other causes in more detail; however, the results are consistent with existing studies that have used mixed-age community-based samples, and have shown that depression and anxiety disorders are associated with multiple causes of death, including diseases of the metabolism, respiratory and nervous systems, and external causes such as accidents and suicide.^9^^,^^33^

A key criticism of existing literature examining affective symptoms and mortality is a general failure to account for potential confounding with concurrent physical and mental health.^34^ Schizophrenia and adolescent externalising behaviour have demonstrated strong associations with premature mortality in the NSHD^23^ and elsewhere;^12^^,^^35^ however, our results were not attenuated by controlling for externalising behaviour, nor were they explained by excluding people with schizophrenia. Likewise, we found little evidence that the associations between affective symptoms and mortality were due to confounding with poor physical health, as controlling for factors relating to poor physical health in childhood, including hospital admissions, sickness absence and birth weight, did little to attenuate associations.

### Strengths and limitations

Methodological strengths of our study include an exceptionally long follow-up period and prospectively obtained data on a wide range of potential confounding variables, although the possibility of residual confounding cannot be excluded. Loss to follow-up was particularly low as there were only 49 study members with non-linked mortality data, and missing covariate data were handled with multiple imputation. Our measure of affective symptoms strongly predicts adult mental disorder in the NSHD,^6^ suggesting good construct validity; furthermore, teacher-rated data have been shown to better predict psychiatric disorder than self-report data in other measures of child and adolescent emotional problems, as shown by the Strength and Difficulties Questionnaire.^36^

Limitations of this study include low power to detect small effects, and so it was difficult to draw strong conclusions about moderate symptoms and estimates regarding cause-specific mortality. Also, the generalisability of the study may be limited because of differences in social and health challenges faced by study members compared with today's children; for example, study members grew up in a post-war economy and in the era of serious childhood diseases such as polio and measles, which have since been effectively eradicated. Likewise, there have been considerable improvements in mental health awareness and treatment since the 1960s, which may buffer the effect of mental health problems on educational and behavioural outcomes associated with mortality; nevertheless, access to adolescent mental health services remains poor, with only around a quarter of young people in the UK getting the help they need.^37^

In conclusion, we have shown that severe adolescent affective symptoms are associated with premature mortality irrespective of a range of potential confounders, including childhood social disadvantage, childhood illness and other mental health problems. Remarkably, the effect of severe adolescent affective symptoms on mortality persisted for the duration of follow-up – over 50 years; however further research is needed to elucidate the role of accumulation and potential mediating mechanisms. There was suggestive evidence of an association between affective symptoms and mortality across multiple causes of death, especially other causes, which warrants more detailed investigation with larger samples as these causes are rare. Given the prevalence of adolescent affective disorder, our findings highlight the importance of early intervention and treatment to prevent associated declines in mental and physical health, and subsequent early mortality. Early identification could be facilitated by routine assessment of mental health during medical check-ups or at school, and enhanced mental health training for health and education professionals.

## Supplementary material

For supplementary material accompanying this paper visit https://doi.org/10.1192/bjp.2018.90. click here to view supplementary material
