# Supplementary material for: Adolescent affective symptoms and mortality
Source: Br J Psychiatry. 2018 Jul;213(1):419–24. doi: 10.1192/bjp.2018.90 (PMC6112411; doi:10.1192/bjp.2018.90)
Supplement: Supplementary file 1 [file S0007125018000909sup001.zip › Supplementary tables 1 to 3_CLEAN.docx]

**Supplementary Table 1.** Hazard ratios for the association between affective symptoms at age 13-15 (alternative cut-point) and all-cause mortality (15-67 years), based on 472 deaths and 20 imputations (n=3884)

|  |  | **Hazard ratio  (95% CI)** | **Test for trend (p)** |
| --- | --- | --- | --- |
| **Adjusted for sex** |  |  |  |
| Affective symptoms^a^ | None/mild | 1.0 | <0.01 |
|  | Moderate | 1.12 (0.91-1.38) |  |
|  | Severe | 1.54 (1.22-1.95)** |  |
| **Adjusted for all** | | |  |
| Affective symptoms^a^ | None/mild | 1.0 | <0.01 |
|  | Moderate | 1.09 (0.88-1.34) |  |
|  | Severe | 1.46 (1.15-1.86)* |  |

a. ‘mild or no’ symptoms: 1st to 50th percentile; ‘moderate’ symptoms: 50th to 83rd percentile; ‘severe’ symptoms: 84th to 100th percentile
* P < 0.05
** P <0.001

**Supplementary Table 2.** Hazard ratios for the association between affective symptoms at age 13-15 and all-cause mortality (15-67 years), based on 374 deaths and study members complete on all covariates (n=3072)

|  |  | **Hazard ratio  (95% CI)** | **Test for linear trend (p)** |
| --- | --- | --- | --- |
| **Adjusted for sex** |  |  |  |
| Affective symptoms | None/mild | 1.0 | <0.01 |
|  | Moderate | 1.17 (0.94,1.46) |  |
|  | Severe | 1.78 (1.30,2.42)** |  |
| **Adjusted for sex, childhood social class** | | |  |
| Affective symptoms | None/mild | 1.0 | <0.01 |
|  | Moderate | 1.14 (0.92,1.42) |  |
|  | Severe | 1.68 (1.23,2.29)* |  |
| **Adjusted for sex, cognition age 8** | | |  |
| Affective symptoms | None/mild | 1.0 | <0.01 |
|  | Moderate | 1.14 (0.91,1.42) |  |
|  | Severe | 1.61 (1.17,2.21)* |  |
| **Adjusted for sex, externalising age 13-15** | | |  |
| Affective symptoms | None/mild | 1.0 | <0.001 |
|  | Moderate | 1.20 (0.96,1.50)) |  |
|  | Severe | 1.84 (1.35,2.51)** |  |
| **Adjusted for sex, birthweight** | |  |  |
|  | None/mild | 1.0 | <0.01 |
|  | Moderate | 1.17 (0.94,1.46) |  |
|  | Severe | 1.78 (1.31,2.43)** |  |
| **Adjusted for sex, sickness absence age 6-10, hospitalisations ages 0-6, 6-10 and 11-15 years** | | |  |
| Affective symptoms | None/mild | 1.0 | <0.01 |
|  | Moderate | 1.14 (0.92,1.43) |  |
|  | Severe | 1.61 (1.17,2.20)* |  |
| **Adjusted for all** | | |  |
| Affective symptoms | None/mild | 1.0 | <0.01 |
|  | Moderate | 1.14 (0.92,1.43) |  |
|  | Severe | 1.57 (1.14,2.17)* |  |

* *P*< 0.05
** *P*<0.001

**Supplementary Table 3.** Hazard ratios for the association between affective symptoms at age 13-15 and cancer and cardiovascular mortality (15-67 years), based on 20 imputations (n=3884)

|  |  | **Mortality hazard ratio (95% CI)** | |
| --- | --- | --- | --- |
|  |  | **Cardiovascular disease**  **(117 deaths)** | **Other causes^b^**  **(91 deaths)** |
| **Adjusted for sex** |  |  |  |
| Affective symptoms | None/mild | 1.0 | 1.0 |
|  | Moderate | 1.24 (0.83-1.84) | 1.55 (0.99-2.44) |
|  | Severe | 1.92 (1.11-3.32)* | 2.65 (1.47-4.80)* |
| **Adjusted for sex, childhood social class** | | |  |
| Affective symptoms | None/mild | 1.0 | 1.0 |
|  | Moderate | 1.18 (0.80-1.75) | 1.53 (0.97-2.41) |
|  | Severe | 1.76 (1.01-3.06)* | 2.62 (1.44-4.78)* |
| **Adjusted for sex, cognition age 8** | |  |  |
| Affective symptoms | None/mild | 1.0 | 1.0 |
|  | Moderate | 1.17 (0.79-1.74) | 1.48 (0.94-2.33) |
|  | Severe | 1.61 (0.92-2.82) | 2.31 (1.26-4.24)* |
| **Adjusted for sex, externalising age 13-15** | | |  |
| Affective symptoms | None/mild | 1.0 | 1.0 |
|  | Moderate | 1.27 (0.86-1.88) | 1.58 (1.00-2.49) |
|  | Severe | 2.00 (1.16-3.45)* | 2.72 (1.50-4.95)* |
| **Adjusted for sex, birthweight** | |  |  |
|  | None/mild | 1.0 | 1.0 |
|  | Moderate | 1.23 (0.83-1.83) | 1.54 (0.98-2.42) |
|  | Severe | 1.90 (1.10-3.29)* | 2.61 (1.45-4.71)* |
| **Adjusted for sex, sickness absence age 6-10, hospitalisations ages 0-6, 6-10 and 11-15 years** | | |  |
| Affective symptoms | None/mild | 1.0 | 1.0 |
|  | Moderate | 1.22 (0.82-1.80) | 1.47 (0.93-2.31) |
|  | Severe | 1.82 (1.06-3.16)* | 2.21 (1.20-4.05)* |
| **Adjusted for all^a^** | | |  |
| Affective symptoms | None/mild | 1.0 | 1.0 |
|  | Moderate | 1.16 (0.79-1.72) | 1.44 (0.91-2.27) |
|  | Severe | 1.65 (0.93-2.91) | 2.03 (1.07-3.85)* |

a. adjusted for sex, childhood social class, cognition age 8, externalising age 13-15, birthweight, sickness absence age 6-10, hospitalisations ages 0-6, 6-10 and 11-15 years
b. diseases of the respiratory system (33%), digestive system (23%), nervous system (16.5%), and all other causes (27.5%)
* P< 0.05
