# Supplementary figures and images for: Adolescent affective symptoms and mortality
Source: Br J Psychiatry. 2018 Jul;213(1):419–24. doi: 10.1192/bjp.2018.90 (PMC6112411; doi:10.1192/bjp.2018.90)

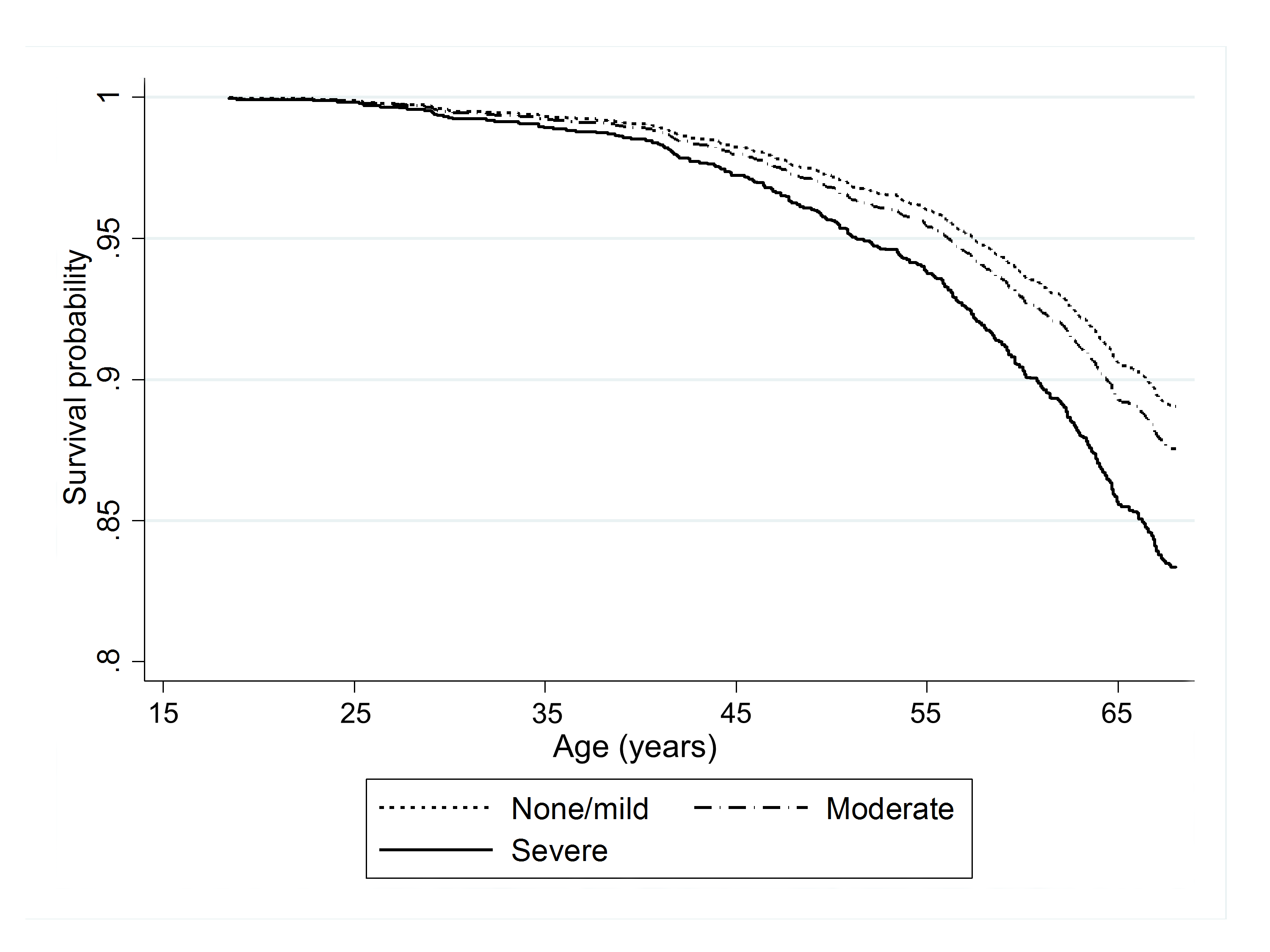

Supplement: Supplementary file 1 [file S0007125018000909sup001.zip › Supplementary figure 1.tif]
